# Annotations

**Published:** 1906-12-15

**Authors:** 


### ANNOTATIONS.

### "Lock-jaw" and Nervous Shock.

The New York correspondent of the *Daily Telegraph* gives a graphic description of how a lady developed a fatal attack of lockjaw as a result of the shock she received from being conveyed at a high speed from the top to the bottom of a twenty-story skyscraper. It is conceivable that a severe condition of nervous prostration might follow such an event, but it would be interesting to know what is meant in this case by the term "lockjaw." Lockjaw is generally understood to be the popular term for the disease known as tetanus, resulting from infection with the tetanus bacillus, and it is difficult to understand how any nervous shock could be made responsible for the introduction of this organism into the system.

### The Vision of the Motorist.

The large number of accidents occurring in our busy thoroughfares testifies to a lack of adjustment between the increased speed and quantity of the traffic and the individual's ability to cope with it, by controlling or avoiding it. A proper acuteness of the senses of sight and hearing is essential in order that anyone may travel on the King's highway without danger to himself or the rest of the community. A properly adjusted vision is above all of the greatest importance to the motor driver. He must be able to judge accurately of distance and speed, and any miscalculations may easily lead to disaster. Mr. Cecil Clements has recently drawn attention to this necessity for visual accuracy, and is disposed to ascribe many motor accidents to errors of vision. In fact, he cites several cases in which defective vision has actually been discovered in the motor driver, after an accident. He rightly points out that motor driving necessitates an alertness which must cause considerable eye and nerve strain, and in this way an error of vision may easily be greatly exaggerated. He suggests that all would-be motor drivers ought to produce satisfactory evidence of good vision before being granted licences.

### Milk Supply.

At the instigation of Dr. J. Mitchell Wilson, Medical Officer of Health for East Riding, a joint committee representing the East and West Ridings of Yorkshire, and the county boroughs of Bradford, Hull, Leeds, Rotherham, and Sheffield, intend to appoint for one year a bacteriologist, to carry out investigations, with a view to ascertain the amount of contamination that milk undergoes, at the different stages in its passage from the cow to the consumer. Dr. Wilson rightly argues, that in the summer, when cows are kept and milked in the open fields, contamination at the farms and during milking should be less likely, whereas infantile diarrhœa is much more common. On the other hand, it is known that the contamination of milk is greatly increased in its transit from the farm to the consumer. This has already been well shown in the report of Dr. Sidney Davies, Medical Officer of Health for Woolwich, on his inspection of the Wiltshire farms, which provide more than half the milk of the borough. Although he finds much to be desired in the arrangements of the farm to ensure cleanliness, and prevent contamination, he is of opinion, that, the depots for receiving the milk at the railway stations are even more responsible for the impurities, which find their way into the milk. The sheds are freely exposed to the open air, and no provision is made to prevent contamination of the milk from the dust of the road, or the breath, hands, or clothes of those entering the depot. The results of the investigation, now to be undertaken in Yorkshire, will be anxiously awaited by all interested in a proper supply of pure milk to the public, and, it is hoped, will lead to more stringent measures to ensure this end.

### Christmas Digestives.

Before the introduction of wines or spirits people had to depend upon home-brewed ale. As new beverages were discovered in the process of civilisation the older customs which connect ale with Christmas feasts gave place—except in the case of those who have a strong reverence for antiquity—to those beverages which had by this time become more fashionable. Instead of the homely rhubarb wine, cowslip wine, or elderberry wine, with which the feast was crowned in early times, and as it still is in homely districts, Christmas came to be connected with digestives of a more potent character. Gin, rum, whisky, port wine, and other spirituous liquors came to be used. Jugged hare was flavoured with port wine; the plum-pudding (symbolical throughout the world of the richness of English hospitality) was mixed with libations of old ale, or, as modern preferences lead, with a quantum of Jamaica rum. More than mere flavouring is required. The demon of indigestion, which is apt to haunt the national dish, is consumed in the flames of burning brandy in which the symbolic pudding is served. Should, perchance, the ghost of this demon still haunt the jovial Christmas feaster, that will quickly vanish under the benignant exorcism of a liqueur. Most people imagine that the ladies of our ancestral homes of England knew less about digestion than the "new woman." They, however, devoted more personal attention to the art of cooking and to the prevention of illness than modern ladies do. On the feast day the lady knew the consequences of her tasty and well-cooked dishes, and understood full well how to divert the disastrous results of an over-enjoyed meal. Experience had taught them that ale and apples tended to promote digestion; that their cordials stimulated circulation of blood in the stomach; that their juleps would prevent flatulence and undue distension; and that by the helpful administration of these things in the midst of their social enjoyments much subsequent discomfort could be avoided.

